# No vaccine against HIV yet-are we not perfectly equipped?

**DOI:** 10.1186/1743-422X-3-60

**Published:** 2006-08-29

**Authors:** Mahender Singh

**Affiliations:** 1 Department of Pathology and Cell Biology, Thomas Jefferson University, Philadelphia, Pennsylvania, USA

## Abstract

Enormous effort has been devoted to the development of a vaccine against human immunodeficiency virus (HIV). But it is proving to be an unprecedented challenge to create an effective vaccine mainly due to the high genetic variability of the virus and the necessity of cytotoxic T lymphocytes (CTL) for containing the infection. Currently pursued vaccine strategies appear to induce CTL in nonhuman primate models but in the early clinical trials, these strategies fail to fully control the viral infection. New strategies that can cover the vast genetic diversity of HIV are needed for the development of a potent vaccine.

## Background

Since it was first reported in 1981, the disease has been misrepresented in mass-media as gay scourge, drug-user's Black Death, a punishment on sinful, etc. The list of stigma goes on mainly due to the unique biology of the causative agent which spreads both venereally and by contaminated blood products. The disease is caused by a retrovirus of the Lentivirus genus under the name of Human Immunodeficiency Virus (HIV-1). Once in the human body, the virus replicates mainly in CD4^+ ^lymphocytes and leads to a progressive degenerative immune deficiency disease, known as acquired immunodeficiency syndrome (AIDS). In just over two decades the virus has killed more than 20 million humans and infected over 42 million people globally with the latest yearly infection rate of over 6 million [1]. Considering the magnitude of the HIV/AIDS epidemic, the efforts in fighting the disease have been extraordinary through developing therapies and potential vaccines. The literature is full with publications and reviews on the subject. Even a deadline has been suggested by President Clinton in 1997 to develop a vaccine by 2007.

In its 2004 report, the AIDS Vaccine Advocacy Coalition (AVAC) documented that there will not be a safe and effective vaccine in 2007 and that we need to "focus on the long haul and set an agenda for sustained and sustainable action that stretches well beyond 2007" [2]. The problem is further compounded by the emergence of drug-resistant variant strains that makes one ask the question: is the replication machinery of HIV so unique that it can easily find a way to evade the therapeutic and preventive approaches, thus, making it difficult to develop a preventive measure against HIV/AIDS? In the following sections I am looking into the unique biology of HIV infection as an impediment to the preventive efforts against HIV/AIDS and also into the possible strategies to overcome such obstacle for developing a vaccine. This article is not intended to be an exhaustive review of research articles on HIV vaccine development. It summarizes the difficult aspects of HIV vaccine development and discusses prospects of novel vaccination strategies.

### Uniqueness of HIV-1 infection

**Figure 1:**
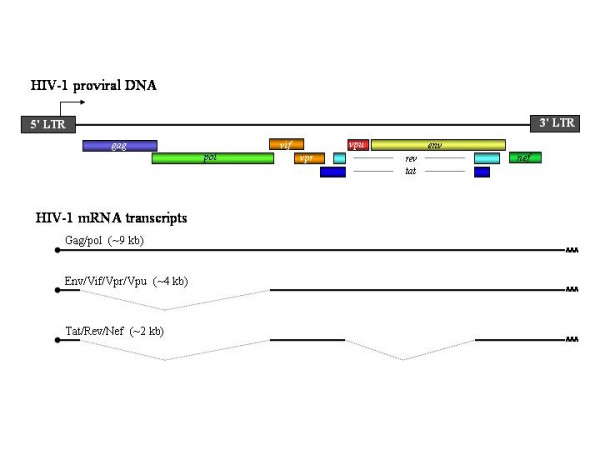
Genome organization of HIV-1. The open reading frames for various polypeptides are shown as rectangles and the transcription initiation site as an arrow. Multiply-spliced mRNA transcripts encoding various proteins are shown with splice-sites together with 5'-cap and 3' polyA tails. Major translated polypeptides from these mRNAs are finally processed to produce 15 protein molecules.

With a genome of approximately nine thousand nucleotides, HIV-1 has packaged the necessary information in overlapping open reading frames to encode 15 proteins from multiply-spliced mRNAs (Figure 1) that provide the unique characteristics to its infection. HIV establishes infection (especially in CD4+ T lymphocytes) by integrating its genome into the host cell genome. The virus spreads by either venereal contact, direct injection of contaminated blood products in the hematogenous circulation or from mother to child during pregnancy or birth. Therefore, any vaccine to be effective must induce mucosal immunity to prevent venereal spread, and the systemic immunity to control the other modes of transmission. A successful vaccine would also be expected to stimulate innate immune system, generate high titers of neutralizing antibodies and strong cellular immune responses leading to persistent and broad spectrum immunity to cover all subtypes of HIV. The initial burst of virus replication following the exposure appears to be contained by a partial antiviral immune response, which is not yet fully characterized. Despite this initial immune response, HIV continues to replicate persistently in infected individuals. The persistent replication in the presence of an immune response and integration of its genetic material in the host genome are the most troubling aspects of HIV-1 biology for developing a vaccine.

Additionally, the replication machinery of the virus is so inaccurate that it generates new mutants for virtually every virion produced in an infected individual, thus, creating a myriad of new and unique viral particles every day [3]. A high number of recombination events occurring during the replication further compounds the genetic heterogeneity. It is this genetic diversity that also accounts for the distinct subtypes or clades of HIV occurring in geographically distinct regions of the globe: for example, clade B viruses cause AIDS epidemic predominantly in the Western Hemisphere, clade C viruses in the sub-Saharan Africa and clade B, C and E in Asian countries. The extraordinary genetic variations create a heterogeneous virus population, often termed as "swarm" or "quasi-species" in an infected individual, which continually supplies new antigenic variants against which no immune response has yet been developed. The mutant viruses keep continually damaging or killing the cells of the immune system (mainly CD4^+ ^lymphocytes) and, thus progressively destroy the body's ability to fight opportunistic infections and certain cancers resulting in AIDS and finally death in 7 to 10 years.

The evolution of HIV is also believed to be the result of genetic heterogeneity. A large number of lentiviruses exist in African nonhuman primates as apathogenic species-restricted simian immunodeficiency viruses (SIV) [4]. Wild populations of chimpanzees are infected with HIV-like viruses which appear to have evolved through recombination of distinct SIV isolates [5] and have zoonotically infected humans to cause the AIDS epidemic [6]. SIV from African monkeys also cause AIDS-like disease in Asian macaques, which are used as nonhuman primate models for understanding viral pathogenesis and evaluating vaccine strategies against HIV [7].

As mentioned above, a potent defense against HIV would require both arms of the immune system: humoral and cellular immunity. The protective role of HIV-neutralizing antibodies in natural infection seems to be insufficient since such antibodies are detected only after several weeks of initial containment of virus replication. Moreover, only low titers of neutralizing antibody are detected in HIV-1 infected individuals. Cellular immune responses seem to have a dominant role in HIV-1 containment as evidenced by several *in vivo *and *in vitro *observations: the emergence of HIV-specific CD8^+ ^CTL responses coincides with the initial containment of viral replication in acute infections [8]; high levels of HIV-specific CTL in the peripheral blood of infected individuals are predictive of good clinical status, measured by plasma viral RNA loads [9]; *in vitro *replication of HIV-1 in CD4^+ ^lymphocytes can be inhibited by CD8^+ ^lymphocytes possibly through direct cytotoxicity and other soluble factors including beta chemokines [10,11]. The most compelling evidence of the importance of CD8^+ ^lymphocytes in controlling HIV replication came from animal models. Monkeys depleted of CD8^+ ^lymphocytes by administering anti-CD8 monoclonal antibodies were unable to control viral replication upon infection with SIV. These animals died of AIDS-like disease with an accelerated course [12].

Mutations have been shown to help HIV escape recognition by CTL [13]. Escape variants happen to be the cause of an abrupt increase in viral replication and decreased immune function in infected individuals. The daunting challenge is to devise an immunogen that can induce high-frequency CTL and antibody responses, which are capable of neutralizing a variety of HIV isolates.

### Failure of traditional preventive approaches

**Figure 2:**
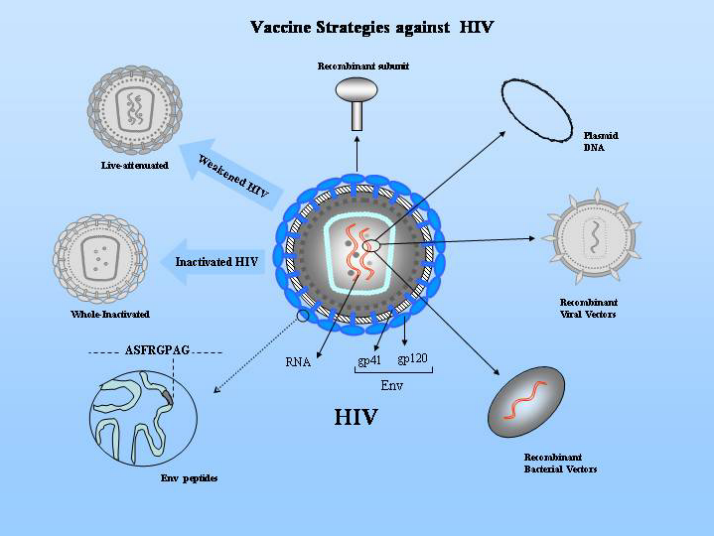
Some of the vaccine strategies against HIV currently under investigation are shown. The HIV virion with RNA and envelope (Env) glycoproteins gp41 and gp120 is also shown.

Traditional strategies for vaccination such as attenuated- or inactivated-viruses, passive immunization and purified or recombinant proteins (Figure 2) safely protect humans against a variety of viral pathogens such as smallpox, measles, polio, rabies, hepatitis B virus, etc. These approaches are not proving useful against HIV-1 due to the unique biology of the infection and failure in eliciting potent immune responses. A detailed overview of various vaccine approaches has been compiled elsewhere [14].

In SIV-macaque models, gene-deleted SIV known to be pathogenically attenuated were found to cause disease in monkeys [15] and the degree of protection was found to be inversely related to the level of attenuation [16]. Similarly, humans who received blood products infected with an HIV-1 isolate harboring a large genetic deletion appeared initially to be free of disease but later developed AIDS [17]. The animal models show that an attenuated virus confers protection only if it can replicate at low but consistent levels. However, even the low level of replication over prolonged periods might afford the virus time to mutate and revert to pathogenic variants. The safety concerns over this modality killed the enthusiasm among investigators for pursuing it as a vaccine approach. Furthermore, chemically inactivated virus vaccines have induced effective immunity in monkeys against SIV [18]. However, this approach is very restricted in duration and spectrum of immune response and fails to induce immunity against genetically diverse viral isolates. Inactivated vaccines also fail to generate CTL responses, thus, there is little optimism that this approach will prove to be useful. Nevertheless, non-infectious particle immunization strategies are being pursued with the expectation that these virus-like particles can be easily manipulated and are safer than inactivated virus. Passive immunization studies, mainly conducted in animal models, have not been encouraging. Trkola *et al. *[19] evaluated the efficacy of passively transferred neutralizing monoclonal antibodies (2G12, 2F5 and 4E10) in suppressing viral rebound in individuals undergoing interruption of antiretroviral therapy. Such an approach would help prolong the life of infected individuals but mass production of high-titer monoclonal antibodies against variant strains may not be a cost effective approach. Finally, highly purified viral proteins expressed in mammalian or bacterial cells using recombinant DNA technologies fail to induce CTL responses or any immunity against genetically diverse HIV isolates. Efficacy trials of such vaccines conducted in United States and Thailand showed no protection against HIV-1 [20]. The failure of traditional approaches asks for exploring novel vaccine strategies against this virus. However, neutralizing monoclonal antibodies against HIV hold some promise and their importance is discussed in the following chapter.

### Prospects of novel vaccination strategies

Live recombinant viral and bacterial vectors and plasmid DNA have been explored as novel approaches for delivering HIV proteins as immunogens (Figure 2). Results of several exciting studies in animal models employing these novel approaches have been reviewed elsewhere [14]. The plasmid DNA is known to be less immunogenic, particularly in inducing CTL, in clinical testing in humans than in animal models. Several improvements such as codon-optimization for expression of viral proteins in mammalian cells, alteration in regulatory elements, inclusion of cytokine expressing genes and novel formulations with polymers are being pursued to increase immunogenicity of DNA vaccines.

Genes of HIV and SIV have also been expressed in microorganisms that have a proven record of being safe and effective live-attenuated vaccines. A long list of such live recombinant vectors includes attenuated vaccinia and other pox-, alpha-, adeno- and measles viruses, attenuated mycobacterium Bacille Calmette-Guerin, Salmonella, Shigella and others. Since several of such vectors are replication competent, expression of HIV proteins from them is expected to induce CTL. Many of the vaccine studies combine various approaches in a prime-boost fashion for avoiding immune responses to the vectors. Results of several animal studies using these modalities have been encouraging, but observations in early phase clinical trials in humans have not been promising. Some of the trials were stopped at various stages owing to adverse reactions to the delivering vector or the inability of the expressed immunogen to cover genetically diverse isolates prevalent in the geographical areas. Nevertheless, the outcome of several ongoing clinical trials is expected to deliver the good news about safe vaccine delivery vectors and, if possible, an effective vaccine against a particular strain of HIV-1 [21].

In anther approach, *in vitro *antigen-pulsed dendritic cells (DC) upon re-injection show improvement in cellular immune responses against the same HIV-1 strain [22]. This approach has potential as a therapeutic vaccine for already ongoing HIV infections but is again limited in not inducing immunity against genetically diverse isolates. DC primed with a cocktail of peptides carrying diverse immunogenic epitopes is an exciting avenue of investigation for inducing immunity against heterogeneous strains of HIV-1. Although *ex vivo *loading of DC seems an exciting avenue for individualized therapeutic intervention, the financial cost of such an approach makes it unattractive endeavor for a prophylactic vaccine in developing countries.

Lately, several neutralizing monoclonal antibodies have been reported [23]. The neutralizing antibodies have potential only if they are able to prevent the binding of cell-free HIV virions to the receptor (CD4) and/or co-receptor (CXCR4/CCR5) on the host cells, thus inhibiting the entry of the virus. Two monoclonal antibodies (2F5 and 4E10) have been very recently demonstrated to bind to membrane proximal linear epitopes of gp41 and broadly neutralize HIV across clades [24]. The crystal structure of the epitope-binding site of 4E10 has already been determined [25]. This information is expected to help design right immunogens that would induce 4E10-like neutralizing antibodies and potentially prevent entry of the virus in the host cells, thus halting further replication and transmission of HIV-1.

### A vaccine for beating the genetic heterogeneity and antigenic diversity

The accumulated experience in vaccine development against HIV highlights the challenge in devising an immunogen that can mount a potent immune response against the continuously arising viral variants and the AIDS epidemic. Using geographically prevalent strains or consensus sequences have so far been the strategies for developing vaccines against antigenic variants of HIV-1 [26]. Lately, clinical trials have also been initiated using combinations of HIV-1 candidate vaccines with the idea of combining the antigenic strength of each vaccine against different clades [27]. The outcome of such combo vaccines remains yet to be seen.

Easier said than done, one can think of utilizing the error-prone replication machinery of HIV to generate potential immunogens that would represent all the variants. In this strategy, one would first replace the transcription-transactivator Tat/TAR axis of HIV with controllable transcription regulators and take out other non-structural protein genes such as *nef *in order to weaken the virus. Several investigators have been pursuing the tetracycline/doxycycline-controlled transcriptional regulator (tetO/tTA or tetO/rtTA) systems [28,29]. This system could be used to generate immunogens *in vitro *or *in vivo*. Since the system has also been shown to have background expression [28], its *in vivo *utilization would require enhanced transcriptional control. More stringency could be added to the system by combining it with the tetO silencer (tTS) that would abrogate the background expression or leakiness [30]. The HIV genome also has a size constraint for inserting additional sequences. To circumvent this hurdle, multiple genomes of HIV can be combined in parallel using the drug-controlled transcription-transactivation system, thus compensating for the insert size constraints and bringing the system under stringent control. This way one would expect to switch on or off the HIV replication machinery in a controlled fashion and generate the necessary immunogens for covering the genetic heterogeneity by utilizing the error-prone HIV replication machinery itself. This approach would need thorough investigation first *in vitro *and later in animals using SIV as a model. The major concerns over this approach would be recombination between the multiple genomes of HIV resulting in pathogenic variants. Moreover, if such viruses capture the cellular promoter/enhancer elements, the conditional replication control would be lost resulting in a pathogenic virus.

Alternatively, one can utilize the knowledge of human genome and HIV sequences for creating "swarm or quasi-species" *in compu *by digitally generating sequences of HIV through combining all the possible substitutions at each nucleotide position. The putative immunogens from such sequence combinations would be identified by digitally matching them to the three-dimensional structures of the human MHC molecules (HLA) for the feasibility of CTL epitopes presentable to the immune system. These epitopes would be screened for their relevance to generate CTL *in vitro *against the prevalent HIV strains. A cocktail of such epitopes would be delivered using live-vectors or primed-DC for generating protective immune responses against the genetic variants. Similarly, putative neutralizing antibody inducing epitopes can also be generated utilizing the information on antigen-binding sites of neutralizing antibodies. These designer cocktails can be readjusted through the digital data-base of prevalent variant viral sequences. Studies on representative or "immunogenic consensus sequence" epitopes from multiple viral variants using computer-driven methods are already underway [31]. The major difficulty in this approach could be the enormity of the size of the digital data-base and servers needed to generate and analyze such epitopes *in compu*, and the optimal delivery vehicles needed for the cocktails. With the latest pledge from Microsoft^® ^for helping investigators to devise strategies against HIV [32], the necessary expertise and digital data-base size appear not to be the limiting factors. The expected positive outcomes of various vaccine approaches currently underway make me believe that an optimal delivery vehicle would soon be available. Given the right tools to combat the strength of HIV in generating diversity, a safe and effective vaccine against HIV/AIDS can be devised in the near future.

## Conclusion

A tremendous amount of economic and intellectual effort has already been invested in the pursuit of a vaccine against HIV. The unique biology of HIV replication and high rate of mutations have made it harder than initially believed to come up with a preventive measure against AIDS. With the technological advancements and concerted efforts from the policy makers and investigators, it seems not far when a preventive vaccine would be available against HIV.

## Abbreviations

AIDS Acquired immunodeficiency syndrome

AVAC AIDS Vaccine Advocacy Coalition

CTL Cytotoxic T lymphocytes

DC Dendritic cells

HIV Human immunodeficiency virus

HIV-1 Human immunodeficiency virus subtype 1

HLA Human leukocyte antigen

MHC Major histocompatibility complex

SIV Simian immunodeficiency virus

rtTA reverse tetracycline-controlled transcriptional activator

tetO tetracycline responsive operator sequences

tTA tetracycline-controlled transcriptional activator

tTS tetracycline-controlled transcriptional silencer

## Competing interests

The author(s) declare that they have no competing interests.
